# COMMENTARY ON “FACTORS ASSOCIATED WITH LONG-TERM FUNCTIONAL OUTCOMES AND PARTICIPATION IN PATIENTS WITH COLORECTAL CANCERS”

**DOI:** 10.2340/jrm.v58.45830

**Published:** 2026-05-25

**Authors:** Mahesh alias Vibhav SANZGIRI, Saumya DIXIT

**Affiliations:** 1 College of Occupational Therapy, National Institute of Speech and Hearing, Thiruvananthapuram, Kerala; 2 Palliative Program, Defeat NCD Foundation, Medical Oncology Department, Goa Medical College, Goa, India

*To the Editor*,

I would like to extend my sincere congratulations to the authors for their insightful study published in your esteemed journal (1). This study presents valuable data on community-dwelling colorectal cancer (CRC) survivors, shedding light on the persistent challenges they face, such as fatigue, bowel dysfunction, and pain, as well as their long-term rehabilitation needs.

While the findings of this study are compelling, I would like to offer several points for consideration that may further enhance the clarity and clinical applicability of the research:

1. Prehabilitation Window and Baseline Metrics: The study assesses patient functioning at an average of 2.5 years post-diagnosis. However, the methodology does not appear to account for whether patients underwent targeted prehabilitation prior to their primary resection surgery. Because pre-surgical medical and functional optimization directly impacts postoperative morbidity and long-term functional baselines (2, 3), controlling for prehabilitation interventions would provide a more accurate picture of why certain survivors demonstrated better long-term resilience and ClinFIT scores.
2. Granularity of Clinical Rehabilitation Interventions: The authors utilize a double variable (received vs none) for “inpatient rehabilitation” in their multivariate regression model. While assessing general rehabilitation is helpful, grouping all therapies together limits the ability to draw specific clinical conclusions. A more granular analysis of medically driven interventions such as targeted pelvic floor rehabilitation for the 17% of patients reporting pelvic issues, or specific functional occupational training for stoma management (noted in 36% of participants), would significantly strengthen the study’s clinical utility over a generalized care model.
3. Occupational Disruption in Adolescent and Young Adult (AYA) Cohorts: The study notes a substantial decrease in full-time employment, dropping from 46% pre-diagnosis to 27% at the time of assessment. While older age (> 60 years) was identified as a predictor of poorer functional outcomes and lower community integration, the unique occupational and vocational disruptions faced by younger demographics warrant distinct attention. A closer subgroup analysis of the AYA oncology population within this cohort could reveal specific, unmet vocational rehabilitation needs that differ fundamentally from those of the older, retired demographic.
4. Efficacy of Symptom-Specific Management: The data highlight that fatigue (77%) and pain (42%) are primary drivers of poorer quality of life (QoL) and community integration. Moving forward, it would be highly beneficial to investigate which specific medical rehabilitation strategies (e.g., structured exercise oncology protocols or specific pain management modalities) most effectively mitigate these variables, rather than relying solely on the passage of time.

Building on the foundation laid by this study, I suggest future research focus on the longitudinal outcomes of highly specific, medical rehabilitation pathways, tracking functional status from the point of prehabilitation through to late survivorship. Furthermore, evaluating the effectiveness of these targeted interventions on returning patients to their primary occupations would offer deeper insights into optimizing recovery.

In conclusion, the authors have made an important contribution to the literature on colorectal cancer survivorship and rehabilitation. Their findings are highly relevant to clinical practice, providing valuable insights that can guide the development of more personalized, interdisciplinary treatment strategies for this patient population. I hope that the authors will continue to explore this important area of research, and I look forward to the future publication of their work.

Thank you for considering these comments, and I eagerly await the ongoing academic dialogue in this field.
