# Supplementary material for: COMMENTARY ON “FACTORS ASSOCIATED WITH LONG-TERM FUNCTIONAL OUTCOMES AND PARTICIPATION IN PATIENTS WITH COLORECTAL CANCERS”
Source: J Rehabil Med. 2026 May 25;58:45830. doi: 10.2340/jrm.v58.45830 (PMC13213409; doi:10.2340/jrm.v58.45830)
Supplement: Related texts

# REPLY TO COMMENTARY ON “FACTORS ASSOCIATED WITH LONG-TERM FUNCTIONAL OUTCOMES AND PARTICIPATION IN PATIENTS WITH COLORECTAL CANCERS”

**Authors:** Bhasker AMATYA, Fary KHAN

**Affiliations:** Department of Rehabilitation Services, Peter MacCallum Cancer Centre, Melbourne Victoria, Australia. Current: Department of Physical Medicine and Rehabilitation, Qatar Rehabilitation Institute, Doha, Qatar

*To the Editor,*

We thank the Editor and the correspondents for their constructive comments on our recent publication (1). The points raised offer important perspectives that enhance the clinical discourse on cancer rehabilitation.

1. Prehabilitation Window and Baseline Metrics: Prehabilitation plays a significant role in postoperative recovery and can influence both immediate morbidity and long-term functional trajectories. Our study was a cross-sectional assessment of community-dwelling survivors at an *average of 2.4 years post-diagnosis*, focusing on the survivorship outcomes in a real-world, community-dwelling cohort. Consequently, retrospective data on prehabilitation interventions and baseline preoperative functional status were not captured. Future longitudinal research should incorporate baseline (pre-diagnosis or preoperative) metrics to better delineate the trajectory of resilience and the specific impact of early-phase interventions on longer-term functional outcomes.
2. Granularity of Clinical Rehabilitation Interventions: We appreciate the observation of categorization of rehabilitation exposure in the study. Rehabilitation programmes provided in our institution are individualized based on the patients’ needs and capabilities. We dichotomized the inpatient rehabilitation (received vs none) to allow inclusion in multivariate models while maintaining statistical robustness given the sample size and number of covariates. While this approach provided a broad overview of service utilization, it does not capture the qualitative differences between various therapy modalities, their duration and intensity. Distinguishing discipline-specific or symptom-targeted interventions (e.g., pelvic floor rehabilitation, stoma care training, fatigue management) was beyond the scope of our study. A “one-size-fits-all” generalized model is insufficient. Future studies with larger cohorts should incorporate intervention-level granularity ideally through prospective designs, to better delineate the effectiveness of specific rehabilitation strategies.
3. Occupational Disruption in Adolescent and Young Adult (AYA) Cohorts: We thank the correspondent for highlighting this important dimension. Our findings demonstrate the substantial decline in full-time employment (from 46% pre-diagnosis to 27% at follow-up), underscoring the significant socioeconomic impact of CRC in the survivors. The data identified age > 60 years as a predictor for poorer functional outcomes (regression model: ≤ 60 vs > 60 years). We acknowledge that the vocational needs of younger survivors are different from those of older populations. Subgroup analysis to inform vocational rehabilitation, return-to-work programmes were beyond the scope of this study.
4. Efficacy of Symptom-Specific Management: The identification of fatigue (77%) and pain (42%) as primary drivers of poor functioning, quality of life (QoL), and community reintegration were key findings in this study. However, given the observational and cross-sectional design, the study was not designed to evaluate the effectiveness of specific interventions, so this was beyond the scope of the study. The study findings, though, highlight symptom burden as a critical target for intervention and future research need to investigate the efficacy of targeted interventions for these prevalent issues.

In conclusion, we thank Sanzgiri and Dixit for the opportunity to engage in this academic discourse as it strengthens the evolving field of cancer rehabilitation and personalized, interdisciplinary care for a longitudinal, pathway-based approach to CRC rehabilitation. We agree that future research should incorporate the cancer care continuum (from prehabilitation through acute care to long-term survivorship), and evaluate targeted, discipline-specific rehabilitation interventions.
